# Genome-Based Analysis Reveals the Taxonomy and Diversity of the Family *Idiomarinaceae*

**DOI:** 10.3389/fmicb.2018.02453

**Published:** 2018-10-11

**Authors:** Yang Liu, Qiliang Lai, Zongze Shao

**Affiliations:** ^1^State Key Laboratory Breeding Base of Marine Genetic Resources, Key Laboratory of Marine Genetic Resources, Key Laboratory of Marine Genetic Resources of Fujian Province, Third Institute of Oceanography, State Oceanic Administration, Xiamen, China; ^2^Laboratory for Marine Biology and Biotechnology, Qingdao National Laboratory for Marine Science and Technology, Qingdao, China

**Keywords:** *Idiomarinaceae*, genome, taxonomy, diversity, dDDH, ANI

## Abstract

*Idiomarinaceae* is a family of Gram-stain negative, mesophilic euryhalophiles. To provide a robust framework for the evolutionary and taxonomic relationships of bacteria of this family, we compared herein the genomes of 36 type strains and 43 non-type strains using 16S rRNA gene sequences, core genome based 78 single-copy orthologous proteins, digital DNA-DNA hybridization and average nucleotide identity (ANI) estimation. The 79 bacteria of this family were consistently divided into taxon I, taxon II, and taxon III corresponding to the three genera *Idiomarina, Pseudidiomarina*, and *Aliidiomarina*, which contained 13 putative new genospecies in addition to 35 well-defined species represented by each type strain. Furthermore, genetic diversity of this family was evident at the genus- and species levels, and exceeded that which is defined currently by the named species. In view of multiple genotypic characteristics clearly distinct from the other two genera, we propose reinstating the genus *Pseudidiomarina* as a monophyletic taxon. Taken together, this is the first genome-based study of the taxonomy and diversity of bacteria within the family *Idiomarinaceae*, and will contribute to further insights into microbial evolution and adaptation to saline environments.

## Introduction

The family *Idiomarinaceae*, belonging to the order *Alteromonadales* within the class *Gammaproteobacteria*, was first introduced by Ivanova et al. (2004) and initially contained only the genus *Idiomarina* (Ivanova et al., 2000). Soon after, the second genus *Pseudidiomarina* was described on the basis of multiple features clearly distinct from bacteria of the genus *Idiomarina* (Jean et al., 2006). However, as more species were identified, the members of the genus *Pseudidiomarina* were reclassified in the genus *Idiomarina* in consideration of reduced discrepancies between the two genera (Taborda et al., 2009). More recently, a novel genus *Aliidiomarina* of this family was proposed by Huang et al. (2012). As a result, the family *Idiomarinaceae* currently embraces the two genera of *Idiomarina* and *Aliidiomarina*; the former contains 27 species with validly published names and the latter contains eight validly named species (List of Prokaryotic names with Standing in Nomenclature^1^), along with the effectively but not yet validly named species *Aliidiomarina haloalkalitolerans* (Srinivas and Anil Kumar, 2012). The members of this family are Gram-stain negative, mesophilic, non-sporulating rods with slightly curved morphology, and require NaCl for growth with the exception of *Idiomarina xiamenensis* (Wang et al., 2011; Albuquerque and da Costa, 2014). Almost all organisms of this family thrive in various habitats with a wide range of salinities, such as coastal and oceanic waters and sediments, solar salt-making works, saline-alkaline soil, inland hypersaline wetlands, and even submarine hydrothermal fluids, some with salinities higher than the surroundings, clearly indicating that they are typical euryhalophiles (Albuquerque and da Costa, 2014). Some bacteria of this family are well-known for distinctive features such as a primary dependence on amino acid catabolism for carbon and energy rather than on sugar fermentation (Hou et al., 2004); the production of diverse enzymes including lipase (Li et al., 2014), serine hydroxymethyl transferase (Kumar et al., 2018), *N-*acetylneuraminate synthase (Garcia Garcia et al., 2015), *etc*.; the synthesis of many kinds of nanoparticles based on selenium (Srivastava and Kowshik, 2016) and lead sulfide (Srivastava and Kowshik, 2017); and the production of Ca–Mg kutnahorite and struvite (Gonzalez-Munoz et al., 2008).

The small subunit ribosomal RNA gene (16S rRNA) has been successfully used for taxonomic classification and in analyses of community structure of Bacteria and Archaea at different taxonomic levels for over 30 years (Yarza et al., 2014). Inevitably, 16S rRNA gene analyses have also played an important role in determining the taxonomy of the family *Idiomarinaceae* (Poddar et al., 2014). On one hand, the establishment of this family relied mainly on the signature nucleotides of the 16S rRNA gene, including the position 143 (C), 662 (A), 682 (A), 830 (T), 856 (A) (Ivanova et al., 2004). On the other hand, based on the analysis of 16S rRNA gene sequences, the type strains of this family were classified into the four groups, labeled Group I, Group II, Group III, and Group IV. The first three of these correspond to the genus *Idiomarina*, while the latter corresponds to the genus *Aliidiomarina* (Poddar et al., 2014). However, we found, unexpectedly, that both Group I and Group II within the genus *Idiomarina* were actually monophyletic in multiple 16S rRNA gene trees, suggesting they represented well-defined lineages. As is well-accepted, from a viewpoint of classical taxonomy, a genus can be defined by uniting the strains from one or more species into a monophyletic cluster of a phylogenetic tree based on single or multiple molecular markers (Thompson et al., 2013). Based on the viewpoint above, the genus *Idiomarina* does not seem to meet the definition of a single genus, and thus need to be further investigated. Meanwhile, we further found that with the increase in number of isolated strains, the 16S rRNA gene trees of the family *Idiomarinaceae* presented inconsistent branching patterns and unstable topological structures, with low bootstrap values. Consequently, reliable and high resolution methods are needed for providing a robust taxonomic classification for bacteria of this family.

Presently, the integration of genomic information into microbial systematics, in addition to physiological and chemotaxonomic parameters as taxonomic criteria, is strongly recommended in the post-genomic era (Chun and Rainey, 2014). Numerous genome-based approaches have been developed and applied for species delineation, for example pan-genome analysis (Medini et al., 2005; Beukes et al., 2017), average nucleotide identity (ANI) analysis (Konstantinidis and Tiedje, 2005; Riesco et al., 2018), and digital DNA–DNA hybridization (dDDH) analysis (Meier-Kolthoff et al., 2013; Montero-Calasanz et al., 2017). The concept of the pan-genome was first introduced in 2005, and includes the core genome composed of genes shared by all strains, a dispensable genome made of genes present in a subset of the strains, and finally strain-specific genes (Medini et al., 2005; Vernikos et al., 2015). As there is a high reliability of using the core genome for bacterial taxonomy at the strain, species and genus levels, and even at higher taxonomic levels, the core genome has been used extensively in comparative genomics of multiple taxa (Colston et al., 2014; Sun et al., 2015; Hesse et al., 2018; Lugli et al., 2018). As the name implies, ANI represents the average nucleotide identity of all orthologous genes shared between two genomes (Konstantinidis and Tiedje, 2005). Owing to the high resolution of ANI, it has been enormously useful in defining species of the Bacteria and Archaea with a proposed species delineation threshold of 95–96% (Richter and Rosselló-Móra, 2009; Salvetti et al., 2018). As one of overall genome relatedness indices (OGRI), dDDH has been recognized for its reproducible, fast and easy-to-implement approaches to replace the traditional DDH (Auch et al., 2010), which for the last few decades has been used as the gold standard for prokaryotic species delineation using a 70% threshold (Wayne et al., 1987). However, the taxonomy and diversity of bacteria of the family *Idiomarinaceae* have not been studied yet using genome-based approaches above up to now.

With the rapid development of next-generation high throughput sequencing technologies, dozens of genomes of bacteria in the family *Idiomarinaceae* are currently available in public databases, and thus provide an ideal opportunity for inferring their evolutionary and taxonomic relationships in depth. In the current study, we first sequenced genomes of 27 type strains of the family *Idiomarinaceae*. Then, we applied four different approaches, including 16S rRNA gene sequence analysis, core genome analysis based on multiple single-copy orthologous protein sequences, dDDH analysis and Orthologous ANI (OrthoANI) analysis using USEARCH, to delineate the taxonomic classification and identification of strains within this family using a dataset of 79 genome sequences. The study revealed the feasibility and advisability of classifying the family *Idiomarinaceae* into three genera, and provides a robust and straightforward genome-based framework for bacteria of this family.

## Materials and Methods

### Bacterial Culture and Genome Sequencing

The 27 type strains studied were obtained from multiple culture collections or personal gifts, as listed in **Table 1** and **Supplementary Table S1**. These strains were cultured aerobically on marine agar 2216 to the late exponential phase. Genomic DNA was extracted from each culture by using the SBS extraction kit (SBS Genetech, Co., Ltd., Shanghai, China) following the manufacturer’s instructions. The genomes of these strains were determined on an Illumina MiSeq platform by Shanghai Majorbio Bio-pharm Technology, Co., Ltd. (Shanghai, China). More than 1 Gbp of clean data for each strain were generated, reaching approximately 250-fold coverage depth. The high quality reads were assembled by using the software SPAdes version 3.7.0 with default parameters (Bankevich et al., 2012). The assembled contigs were submitted to the GenBank database and the assigned accession numbers are listed in **Table 1**.

**Table 1:** General feature of 79 *Idiomarinaceae* genomes used in this study.

Genome original name	Taxon	No. of contigs	Genome sizes (Mbp)	G + C contents (mol%)	Accession
*I. abyssalis* UBA4213	I-1	5	2.68	46.98	DFTL00000000–
*Idiomarina* sp. CNP8	I-1	71	2.61	46.95	PBST00000000^∗^
*I. abyssalis* UBA3602	I-1	47	2.69	46.97	DFMD00000000^∗^
*I. abyssalis* UBA5050	I-1	68	2.70	46.95	DIBO00000000^∗^
*I. abyssalis* KMM 227^T^	I-1	12	2.68	47.15	FPBE00000000^∗^
*Idiomarina* sp. SAT134	I-1	70	2.30	47.10	PAWS00000000^∗^
*I. abyssalis* UBA3100	I-1	45	2.66	47.05	DFAS00000000^∗^
*Idiomarina* sp. SP350	I-1	9	2.65	47.06	PBDK00000000^∗^
*I. abyssalis* UBA2690	I-1	7	2.64	47.07	DELS00000000^∗^
*Idiomarina* sp. CPC33	I-1	153	2.06	46.76	NYUW00000000^∗^
*Idiomarina* sp. UBA4206	I-1	78	2.77	47.07	DFTS00000000^∗^
*I. abyssalis* UBA5042	I-1	15	2.76	46.94	DIBW00000000^∗^
*I. abyssalis* UBA3616	I-1	65	2.72	46.91	DFLP00000000^∗^
*I. abyssalis* UBA3366	I-1	29	2.77	47.03	DEQM00000000^∗^
*Idiomarina* sp. EAC55	I-1	34	2.89	46.78	NZQS00000000^∗^
*Idiomarina* sp. SP93	I-1	20	2.74	46.95	PAYT00000000^∗^
*I. abyssalis* UBA1461	I-1	25	2.80	46.99	DCSR00000000^∗^
*I. ramblicola* R22^T^	I-2	16	2.71	46.93	PIQC00000000^§^
*Idiomarina* sp. UBA1919	I-3	57	2.96	46.79	DDFV00000000^∗^
*Idiomarina* sp. UBA3992	I-3	62	2.97	46.78	DGBY00000000^∗^
*Idiomarina* sp. UBA3176	I-3	27	2.93	46.77	DEXU00000000^∗^
*Idiomarina* sp. UBA4211	I-3	163	2.86	46.72	DFTN00000000^∗^
*Idiomarina* sp. CPC80	I-3	101	2.15	47.23	NYSZ00000000^∗^
*I. loihiensis* L2TR^T^	I-3	1	2.84	47.04	AE017340^∗^
*I. loihiensis* GSL 199	I-3	1	2.84	47.04	CP005964^∗^
*Idiomarina* sp. NORP114	I-3	61	2.15	47.11	NVTN00000000^∗^
*I. loihiensis* H2.2	**I-4**	28	3.00	46.94	QGUA00000000^∗^
*Idiomarina* sp. 28-8	**I-5**	110	2.97	46.91	BANL00000000^∗^
*Idiomarina* sp. UBA4520	**I-6**	22	2.47	47.16	DGMK00000000^∗^
*Idiomarina* sp. UBA4519	**I-6**	121	2.51	47.22	DGML00000000^∗^
*Idiomarina* sp. REDSEA-S27_B4	**I-6**	60	2.26	47.20	LUNZ00000000^∗^
*Idiomarina* sp. REDSEA-S21_B4	**I-6**	60	2.26	47.20	LUNZ00000000^∗^
*I. zobellii* KMM 231^T^	I-7	30	2.58	47.08	FNCB00000000^∗^
*Idiomarina* sp. UBA5185	**I-8**	202	2.37	47.15	DHWJ00000000^∗^
*I. piscisalsi* TPS4-2^T^	I-9	24	2.58	47.03	PIQA00000000^§^
*Idiomarina* sp. X4	**I-10**	1	2.62	47.27	CP025000^∗^
*I. piscisalsi* 10PY1A	**I-11**	1	2.59	47.36	CP022133^∗^
*Idiomarina* sp. T82-3	I-12	38	2.72	47.28	LSBQ00000000^∗^
*I. baltica* UBA859	I-12	137	2.40	47.48	DBGH00000000^∗^
*I. baltica* OS145^T^	I-12	70	2.72	47.30	AAMX00000000^∗^
*Idiomarina* sp. UBA3162	**I-13**	108	2.60	47.60	DEYI00000000^∗^
*Idiomarina* sp. MD25a	**I-14**	7	2.68	47.47	MDGT00000000^∗^
*I. fontislapidosi* F23^T^	I-15	47	2.88	47.75	PIPV00000000^§^
*Idiomarina* sp. OT37-5b	I-16	1	2.89	51.06	CP027188^∗^
*I. aquatica* SN-14^T^	I-16	9	2.97	50.99	PIPS00000000^§^
*Idiomarina* sp. NP27	**I-17**	87	3.10	48.07	PBYP00000000^∗^
*I. seosinensis* CL-SP19^T^	I-18	7	2.69	47.27	PIQF00000000^§^
*I. tyrosinivorans* CC-PW-9^T^	I-19	22	2.43	49.25	PIQH00000000^§^
*I. xiamenensis* 10-D-4^T^	I-20	77	2.90	49.48	AMRG00000000
*Idiomarina* sp. NORP21	II-1	41	2.34	47.27	NVXC00000000^∗^
*I. marina* PIM1^T^	II-1	11	2.42	47.17	PIPZ00000000^§^
*Idiomarina* sp. 34-48-12	**II-2**	283	2.10	47.52	NCJS00000000^∗^
*I. tainanensis* PIN1^T^	II-3	6	2.38	47.35	PIQJ00000000^§^
*I. maritima* CGMCC 1.7285^T^	II-3	8	2.44	47.38	FOYU00000000^∗^
*I. woesei* DSM 27808^T^	II-4	16	2.44	47.80	LIPW00000000^∗^
*I. donghaiensis* 908033^T^	II-5	16	2.58	48.07	PIPU00000000^§^
*I. indica* CGMCC 1.10824^T^	II-6	31	2.21	49.30	FMXN00000000^∗^
*I. maritima* 125B1	**II-7**	51	2.78	47.85	QGTT00000000^∗^
*Idiomarina* sp. UBA6142	**II-8**	129	1.82	50.42	DIVC00000000^∗^
*I. salinarum* ISL-52^T^	II-9	6	2.49	52.94	PIQD00000000^§^
*I. homiensis* PO-M2^T^	II-10	6	2.61	49.98	PIPX00000000^§^
*I. aquimaris* SW15^T^	II-11	23	2.80	50.24	PIPT00000000^§^
*I. insulisalsae* CVS-6^T^	II-12	20	2.57	52.33	PIPY00000000^§^
*I. sediminum* c121^T^	II-13	8	2.63	50.35	PIQE00000000^§^
*I. halophila* BH195^T^	II-14	11	2.62	50.58	PIPW00000000^§^
*I. atlantica* G5_TVMV8_7^T^	II-15	49	2.70	50.23	JPIN00000000^∗^
*I. taiwanensis* PIT1^T^	II-16	8	2.20	49.47	PIQG00000000^§^
*I. aestuarii* KYW314^T^	II-17	13	2.65	49.10	PIPR00000000^§^
*I. planktonica* TS-T11^T^	II-18	6	2.58	48.97	PIQB00000000^§^
*Idiomarina* sp. A28L	**III-1**	28	2.59	45.50	AFPO00000000^∗^
*A. shirensis* AIS^T^	III-2	15	2.71	46.34	PIPP00000000^§^
*A. iranensis* GBPy7^T^	III-3	36	2.65	46.80	PIPJ00000000^§^
*A. haloalkalitolerans* AK5^T^	III-4	29	2.68	49.29	PIPI00000000^§^
*A. sanyensis* GYP-17^T^	III-5	48	2.60	50.82	PIPM00000000^§^
*A. taiwanensis* AIT1^T^	III-6	30	2.53	48.66	PIPQ00000000^§^
*A. soli* Y4G10-17^T^	III-7	12	3.10	51.61	PIPO00000000^§^
*A. sedimenti* GBSy1^T^	III-8	10	2.85	52.09	PIPN00000000^§^
*A. maris* CF12-14^T^	III-9	45	3.02	50.10	PIPK00000000^§^
*A. minuta* MLST1^T^	III-10	7	2.96	48.69	PIPL00000000^§^


I, *Idiomarina*; II, *Pseudidiomarina*; III, *Aliidiomarina*. The putative new genospecies are shown in bold. All type strains are marked by a superscripted capital T. The genomes from the GenBank database and this study are marked by ^∗^ and ^§^ after the accession, respectively.

### Genome Collection and Analyses

Seventy nine *Idiomarinaceae* genomes, comprising the 27 obtained by this study and 52 available in the GenBank database, were collected (**Table 1** and **Supplementary Table S1**). The strains used in this study included 36 type strains and 43 non-type strains, many of which have not been classified at the species level. Since most of the genomes analyzed in this study were in draft status, assessment of the genomes was conducted by using the software CheckM version 1.0.9 (Parks et al., 2015), which inspected the existence of gene markers specific to the Gammaproteobacteria (UID4761) lineage. The genome annotations and core genome analysis were performed by using the Pathosystems Resource Integration Center (PATRIC) resource^2^ (Wattam et al., 2014). The 16S rRNA gene sequences and single-copy orthologous protein sequences were obtained from each genome of these strains based on the PATRIC analysis. Pairwise similarities of 16S rRNA genes were calculated by using the software DNAMAN version 7.0 (Lynnon Biosoft, Vaudreuil, QC, Canada) with the distance method of Jukes and Cantor. Statistical analysis of 16S rRNA gene similarities, the G + C contents and the genome sizes was performed using Tukey’s *post hoc* test. In these analyses, the null hypothesis was rejected at the 0.05 level.

### Phylogeny of 16S rRNA Gene and Core Genes

The phylogenetic analyses of 16S rRNA genes and the core genomes were performed as follows. All sequences were aligned by using the software MAFFT version 7.311 with default settings (Katoh and Standley, 2013), and then trimmed to match the length of the shortest. The core genome identified consisted of 78 concatenated protein sequences (**Supplementary Table S2**). The phylogenetic trees of 16S rRNA genes and core genomes were built by using the software FastTree version 2.1.10 using JTT + CAT parameters and 1000 bootstrap replicates (Price et al., 2010). *Escherichia coli* str. K-12 substr. MG1655 (GenBank Accession No. U00096) was used as an outgroup in all phylogenetic analyses. The visualization, annotation and management of the phylogenetic trees were performed by using the web-based tool Interactive Tree Of Life (iTOL)^3^ (Letunic and Bork, 2016). In addition, the analysis of signature nucleotides of the 16S rRNA genes for bacteria within this family was performed by using the software MEGA version 5.05 (Tamura et al., 2011). The locations of signature nucleotides were determined by referring to 16S rRNA gene sequence of *E. coli* str. K-12 substr. MG1655.

### Overall Genome Relatedness Indices Calculations

The Genome-to-Genome Distance Calculator (GGDC) version 2.1 online service was employed to calculate the dDDH values between 79 genomes, applying the recommended formula 2^4^. The OrthoANIu tool was used to calculate the ANI values between these genomes based on the improved ANI algorithm with USEARCH^5^ (Yoon et al., 2017). The visualization of the numerical matrix for dDDH and ANI values was carried out by using the pheatmap R package version 0.7.7.

## Results

### Bacterial Genomes Within the Family *Idiomarinaceae*

A total of 79 bacterial genomes within the family *Idiomarinaceae* were analyzed in this study, including five complete and 74 draft genome sequences (**Table 1** and **Supplementary Table S1**). Based on the estimations from the CheckM, most genomes satisfied the criteria required to be considered a near-complete genome with low contamination (≥90% of completeness value and ≤5% of contamination value) (Parks et al., 2015). The general features of the 79 genomes are summarized in **Table 1** and **Supplementary Table S1**. The genomic G + C contents of bacteria of the family *Idiomarinaceae* ranged from 45.50 mol% (*Idiomarina* sp. A28L) to 52.94 mol% (*Idiomarina salinarum* ISL-52^T^), with a mean of 48.04 ± 1.60 mol%. As shown in **Supplementary Figure S1**, the genomic G + C contents among the eight families of the order *Alteromonadales* present significant differences from each other (indicated by different letters on the boxplots). The estimated genomic sizes ranged from 2.17 Mbp (*Idiomarina* sp. UBA6142) to 3.67 Mbp (*Idiomarina* sp. NP27), with an average of 2.73 ± 0.25 Mbp (**Supplementary Table S1**). Intriguingly, the genomic sizes of the family *Idiomarinaceae* were notably smaller than those of members of the other seven families (**Supplementary Figure S1**), implying that the cosmopolitan bacteria of this family are examples of newly streamlined chemoheterotrophic organisms (Giovannoni et al., 2014).

### 16S rRNA Gene Sequences Analysis

**FIGURE 1:**
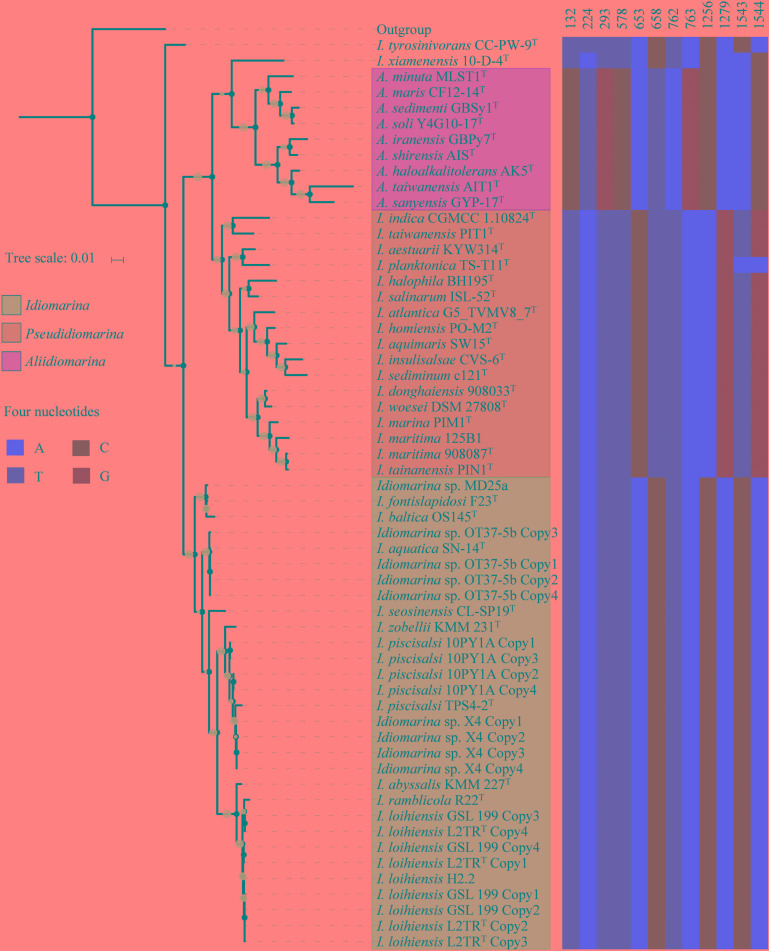
Phylogenetic tree based on 58 of 16S rRNA gene sequences (left panel) and signature nucleotides comparison (right panel) of 42 bacteria within the family *Idiomarinaceae*. The phylogeny was inferred by using FastTree 2.1.10 with JTT + CAT parameters and 1000 bootstrap replicates and rooted by using *Escherichia coli* str. K-12 substr. MG1655. Bootstrap values are indicated on the nodes with different sizes of solid circle filled by light blue. The locations of signature sites were determined by referring to 16S rRNA gene sequence of *E. coli* str. K-12 substr. MG1655. Each of type strains is marked by a superscripted capital T.

**FIGURE 2:**
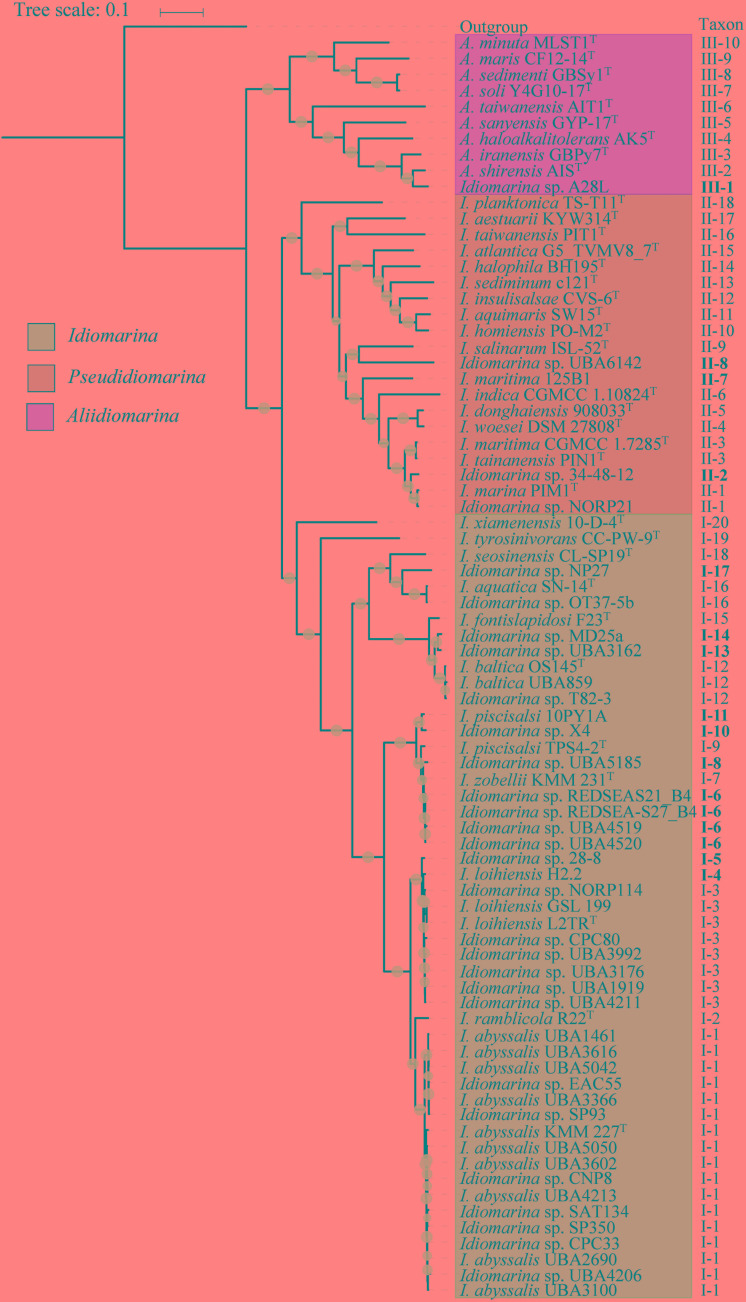
Phylogenetic tree based on core genome of 79 bacteria within the family *Idiomarinaceae*. The phylogeny was inferred by using FastTree 2.1.10 with JTT + CAT parameters and 1000 bootstrap replicates and rooted by using *E. coli* str. K-12 substr. MG1655. Bootstrap values are indicated on the nodes with different sizes of solid circle filled by light blue. The putative novel genospecies are highlighted in bold. Each of type strains is marked by a superscripted capital T.

From 48 bacterial genomes, 63 16S rRNA gene sequences were determined, including 58 complete sequences from 42 bacteria and 5 partial sequences; the 16S rRNA gene was missing from the remaining 31 genomes analyzed (**Supplementary Tables S3, S4**). Interestingly, each complete genome possessed four copies of the 16S rRNA gene, all of which were exhibited intragenomic heterogeneity (**Figure 1**). The 58 complete 16S rRNA gene sequences were used for phylogenetic and similarity analyses, and all 63 sequences for the signature nucleotides analysis. In the 16S rRNA gene tree (**Figure 1**), 42 strains of the family *Idiomarinaceae* were divided into three groups, corresponding to the genera *Idiomarina, Pseudidiomarina* (as originally defined) and *Aliidiomarina*, respectively. On the whole, the phylogenetic relationships of the members of the family *Idiomarinaceae* in the 16S rRNA gene tree (**Figure 1**) are consistent with those in the core genes tree below (**Figure 2**). However, two notable distinctions between the two trees were evident: (1) the two type strains, *Idiomarina tyrosinivorans* CC-PW-9^T^ and *I. xiamenensis* 10-D-4^T^, formed branches separate from the three groups in 16S rRNA gene tree; (2) the two genera *Aliidiomarina* and *Pseudidiomarina* were sister groups in 16S rRNA gene tree, but the genus *Aliidiomarina* was more distant to the genus *Pseudidiomarina* relative to the genus *Idiomarina* in the core genome tree below.

**FIGURE 3:**
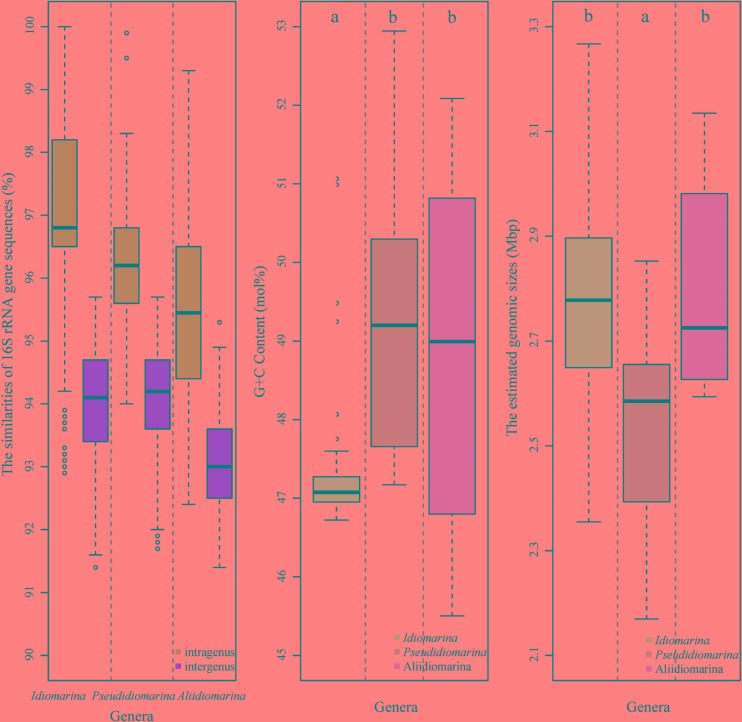
Boxplot of the similarities of 16S rRNA gene sequences **(left)**, genomic G + C contents **(middle)**, and genomic sizes **(right)** of bacteria within the family *Idiomarinaceae*. In the boxplot, the central rectangle spans the first quartile to the third quartile, a segment inside the rectangle shows the median, “whiskers” above and below the box show the locations of the minimum and maximum, and the unfilled circles show suspected outliers. Means with different letters at the top of figure denote significant differences (Tukey’s hsd *post hoc* test, *α* = 0.05; a < b). The estimated genomic size of a bacterium (bp) = sequencing genomic size (bp)/completeness (%).

Based on the phylogenomic analysis below, similarities and signature nucleotide analyses of the 16S rRNA genes were also conducted in this study. The intra-genus and inter-genus means of 16S rRNA gene sequences similarities for the members of the genera *Idiomarina, Pseudidiomarina*, and *Aliidiomarina* were 97.27 ± 1.66 and 93.93 ± 0.95%, 96.28 ± 0.97 and 94.10 ± 0.83%, 95.46 ± 1.59 and 93.06 ± 0.78%, respectively (**Figure 3** and **Supplementary Table S3**). All inter-genus means among the three genera were significantly lower than the threshold value of 94.5% that has been used to define the genus (Yarza et al., 2014), indicating that the genus *Pseudidiomarina* should have been retained as an independent genus, rather than integrating it into the genus *Idiomarina*. As shown in **Supplementary Table S4**, the previously reported family-signature nucleotides and most group-signature nucleotides of the 16S rRNA gene were unsupported and thus should not be used in future studies. As shown in **Figure 1**, 12 newly specific signature nucleotides were useful for differentiating the three genera.

### Phylogenomic Analysis of the Family *Idiomarinaceae*

Prior to the phylogenomic analysis, the G + C contents and estimated sizes of the genomes of members of the three genera were analyzed by using Tukey’s *post hoc* test. As illustrated in **Figure 3**, the genomic G + C contents of members of the genera *Idiomarina, Pseudidiomarina*, and *Aliidiomarina* bacteria were 47.36 ± 0.92, 49.04 ± 1.83, and 49.38 ± 2.00 mol%, respectively, demonstrating significant differences (indicated by different letters on the boxplots, **Figure 3**, the same below) between the genus *Idiomarina* and each of the latter two genera. The estimated genome sizes of members of the three genera above were 2.80 ± 0.23, 2.52 ± 0.19, and 2.80 ± 0.20 Mbp, respectively, revealing obvious contrasts (the same as above) between the genus *Pseudidiomarina* and each of the other two genera.

A total of 78 orthologous protein sequences were used to infer the phylogeny of 79 bacteria of this family (**Supplementary Table S2**). As shown in **Figure 2**, the phylogenetic tree was characterized by high bootstrap values, indicating that the proteins selected were reflective of a robust evolutionary relatedness between bacteria within the family. The trees showed that bacteria of this family were grouped into three monophyletic taxa, matching exactly to the three genera, *Idiomarina, Pseudidiomarina*, and *Aliidiomarina* (defined by the three colors). Taxon I incorporated 49 strains, including 11 type strains and 38 non-type strains. Taxon II contained 20 strains, comprised of 16 type strains and 4 non-type strains, which should be restored or reclassified to the genus *Pseudidiomarina* rather than the genus *Idiomarina*. Taxon III included 10 independent branches corresponding to the nine type strains of the genus *Aliidiomarina* and *Idiomarina* sp. A28L. Taxon I and taxon II exhibited a closer relationship rather than taxon III, and were found to be sister groups. The comparison between the phylogenetic reconstructions based on 16S rRNA genes and the core genome suggested that the core genome provides a more robust phylogenetic framework for this family, and thus can be considered as the preferential approach for genus- and species-wide investigations.

### The dDDH and ANI Analyses for Species Delineation

**FIGURE 4:**
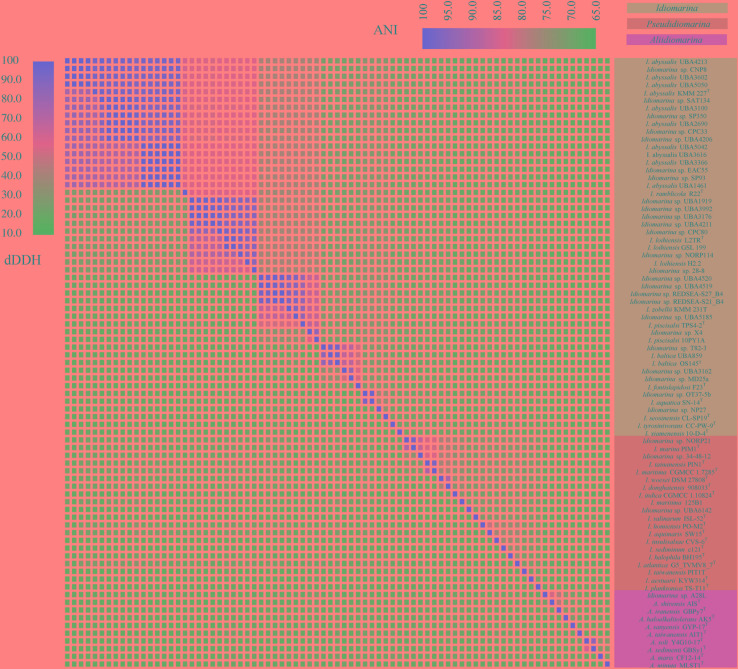
Heatmap of digital DNA-DNA hybridization values in lower-left and average nucleotide identity values in upper right for 79 genomes of bacteria within the family *Idiomarinaceae*. The bacteria of the three genera are shown in the three colors.

The dDDH and ANI are based on the complete genomic information (OGRI), thereby enabling accurate delineation of species and assessment of their genetic relatedness. Therefore, pairwise dDDH and ANI values of the 79 genomes for bacteria of the family *Idiomarinaceae* were calculated and are shown in **Figure 4** and **Supplementary Tables S5, S6**. According to the 70% dDDH (Wayne et al., 1987) and 95% ANI thresholds (Richter and Rosselló-Móra, 2009) for species delineation, the 79 strains fell into 48 genospecies labeled by I-1 to I-20 in taxon I, II-1 to II-18 in taxon II, and III-1 to III-10 in the taxon III, each containing one to 17 strains. Taxon I included nine putative new genospecies (I-4, I-5, I-6, I-8, I-10, I-11, I-13, I-14, and I-17, shown in bold, **Table 1**, the same below) and 11 already described species represented by a type strain. Taxon II consisted of three putative new genospecies (II-4, II-7, and I-8) and 15 validly named species. It was notable that two type strains, *Idiomarina maritima* CGMCC 1.7285^T^ and *Idiomarina tainanensis* PIN1^T^, share a dDDH of 75.8% and ANI of 97.29%, values which are higher than the two thresholds noted above, and therefore they should be considered conspecific. We propose returning these strains to the reinstated genus *Pseudidiomarina* and in this context it is noted that the name *Pseudidiomarina tainanensis* Jean et al. (2009) has nomenclatural priority over *Pseudidiomarina maritima* Wu et al. (2009). Taxon III contained only one putative new genospecies (III-1) and nine well-defined species. Most interestingly, most of new genospecies are distributed in the taxon I corresponding to the genus *Idiomarina*, implying that bacteria of this taxon may possess more novel genotypes relative to those of the two other taxa. In other words, the genus *Idiomarina* possessed higher species diversity, with more new genospecies relative to the other two. Similarly, some species such as I-1 (*I*. *abyssalis*) and I-3 (*I. loihiensis*) possessed higher strain diversity compared with others. This analysis indicated that the genetic diversity of this family varies to some extent at the genus and species levels.

In addition, the species-level phylogenetic classification of 13 other strains of this family was determined by comparing the dDDH values with the 79 strains mentioned above (**Supplementary Tables S1, S7**), although they were not used in the above analysis in view of the low quality of their genomes as assessed by CheckM. As listed in **Supplementary Table S1**, among these strains, six appear to belong to a new genospecies named II-19, three to I-1 and the remaining four to I-8, I-12, I-17, and II-11, respectively. Therefore, based on the OGRI analysis, the proposed species designations of bacteria of this family is shown in **Supplementary Table S1**.

## Discussion

The members of the family *Idiomarinaceae* are ubiquitous in various saline environments, especially in the marine environments (Albuquerque and da Costa, 2014). The widespread distribution of bacteria of this family implies that they may have as yet unexplored diversity. In this report, we performed a genome-based systematic analysis to delineate the taxonomic relationships of 79 strains within the family *Idiomarinaceae*. The results of 16S rRNA gene sequence analysis, the phylogenomic analysis using 78 single-copy core genes, and analysis of two overall genome relatedness indices (dDDH and ANI) were almost completely consistent, and determined the presence of three genus level taxa including 48 genospecies. These results demonstrated that the latter three genome-based methods relative to 16S rRNA gene analysis can provide a more robust taxonomic framework for bacteria of the family *Idiomarinaceae* at the genus and species level.

As mentioned above, the genus *Pseudidiomarina* was introduced to accommodate *Idiomarina*-like organisms without motility, DNAse activity and growth at pHs below 6, and the presence of a unique nucleotide substitution of A instead of C at position 143 in their 16S rRNA gene sequences (Jean et al., 2006). However, due to few distinguishing phenotypic and chemotaxonomic features, except for differences in 16S rRNA gene sequence signature nucleotides, the members of the genus *Pseudidiomarina* were later transferred to the genus *Idiomarina* (Taborda et al., 2009). However, the current study provides an improved taxonomic understanding of this family using genome-based methods. On one hand, the genus *Pseudidiomarina* was found to be an independent monophyletic group in the two phylogenetic trees, meeting with the classical definition of genus (Medini et al., 2005). On the other hand, 16S rRNA gene sequences similarities between the members of the genus *Pseudidiomarina* and the other strains analyzed were lower than the threshold value for delineating a genus (Yarza et al., 2014). In addition, the genomic G + C contents and genome sizes of the members of the genus *Pseudidiomarina* were statistically significant different compared to members of the two other genera in the family. Based on the analyses above, we propose to reinstate the taxonomic status of the genus *Pseudidiomarina*.

Combined with the three genome-based analyses, a considerable diversity of bacteria of the family *Idiomarinaceae* was demonstrated in this study. Among these genospecies clusters of this family, 35 were within the radius of an existing type strain and could thus be successfully linked to a described species. In contrast, the remaining 13 did not contain a described type strain and therefore await formal description and naming in accordance with the rules of nomenclature. In this study, 52 genomes were obtained from the GenBank database, most of which were metagenome-assembled genomes from global ocean samples collected during the Tara Oceans circumnavigation expedition (Parks et al., 2017; Tully et al., 2018). It is notable that all the isolates from the Tara Oceans project fell into taxon I (the genus *Idiomarina*) with the exception of *Idiomarina* sp. NORP21; moreover, they were mainly concentrated in three genospecies clusters (I-1, I-3, and I-6). These results suggested that the strain diversity of the above three taxa is higher than the others, and that the species diversity of the genus *Idiomarina* may be higher than that of the other two genera. When increasing the number of bacteria used to produce a 16S rRNA gene supertree of 717 bacteria, a similar trend of diversity was also observed (**Supplementary Figure S2** and **Supplementary Table S8**). The genetic characteristics and underlying mechanisms of this phenomenon need to be further investigated by comparative genomic analyses in future studies. Moreover, on the basis of this study, a relation between the geographic origin and the genomic relatedness of the strains within this family warrant further investigation.

Over the past few decades, the 16S rRNA gene has been the only widely used molecular marker for prokaryotic taxonomy and is the cornerstone for the current systematic classification of the Bacteria and Archaea (Tindall et al., 2010). However, this marker is incapable of accurately distinguishing closely related strains and species, and of providing the robust phylogenetic relationships of some strains because of its low resolution (Rajendhran and Gunasekaran, 2011; Liu et al., 2013, 2015). In this study, the limitation of 16S rRNA gene analysis was also shown by comparison of the 16S rRNA gene tree and with that derived from the core genome. Moreover, based on the five complete genomes, we found that bacteria of the family *Idiomarinaceae* present heterogeneity in their 16S rRNA gene sequences. For example, Copy 3 of the 16S rRNA gene sequence for *I. loihiensis* L2TR^T^ shares 99.8% similarity with Copy 4. Similar situations have been reported for other strains, species and genera, for example the *Bacillus cereus* group (Liu et al., 2015) and the genera *Pseudomonas* (Bodilis et al., 2012) and *Aeromonas* (Morandi et al., 2005). In contrast, in this study the genome-based phylogenetic analysis and relatedness indices analysis present an improved picture of the phylogeny of the family *Idiomarinaceae* relative to 16S rRNA gene. Thus, based on the current study and previous reports, we strongly recommend that the taxonomy and systematics of Bacteria and Archaea should take full advantage the increasing availability of genomic information (Yarza et al., 2013) and the inherent strength of genome-based approaches (Yarza et al., 2013; Chun et al., 2018; Rodriguez-R et al., 2018).

## Conclusion

The family *Idiomarinaceae* is more diverse at the genus and species level than was previously known and comprises of three genera, in which we have identified 13 potentially novel genospecies in addition to the 35 already described species. Furthermore, the taxonomic status and correct name of strains within this family can be determined based on the genomic analyses. The study also reveals that the genus *Pseudidiomarina* should be reinstated in view of its distinctive phylogenetic divergence and other differences, as originally discussed (Jean et al., 2006). This study structures a robust taxonomic framework of currently available *Idiomarinaceae* strains, and provides an avenue to a better understanding of the functional adaptation to saline environments.

### Taxonomic Consequences

Based on phenotypic and genotypic data, we propose the reclassification of *Idiomarina insulisalsae* as *Pseudidiomarina insulisalsae* comb. nov., *Idiomarina aquimaris* as *Pseudidiomarina aquimaris* comb. nov., *Idiomarina indica* as *Pseudidiomarina indica* comb. nov., *Idiomarina planktonica* as *Pseudidiomarina planktonica* comb. nov., *Idiomarina woesei* as *Pseudidiomarina woesei* comb. nov., *Idiomarina halophila* as *Pseudidiomarina halophila* comb. nov. and *Idiomarina atlantica* as *Pseudidiomarina atlantica* comb. nov.

### Emended Description of the Genus *Pseudidiomarina* (Jean et al., 2006)

*Pseudidiomarina* (Pseud.i.di.o.ma.ri’na. Gr. adj. *pseudes* false; N.L. fem. n. *Idiomarina* a name of a bacterial genus; N.L. fem. n. *Pseudidiomarina* false *Idiomarina*).

The original description is based on that given by Jean et al. (2006). Members are Gram- stain negative rods belonging to the family *Idiomarinaceae* within the class *Gammaproteobacteria*. Some strains are motile by means of a single polar flagellum or peritrichous flagella, while some are non-motile. All strains are chemoheterotrophs, positive for oxidase and catalase, and strictly aerobic with the exception of the catalase negative and facultatively anaerobic *Pseudidiomarina sediminum*. Molecular oxygen is a universal electron acceptor while in some cases nitrate can be used as an alternative electron acceptor under anaerobic conditions. The major fatty acids are iso-C_15:0_, iso-C_17:0_ and Summed feature 9 (iso-C_17:1_*ω*9*c* and/or C_16:0_ 10-methyl). The major polar lipids are phosphatidylethanolamine (PE), phosphatidylglycerol (PG), and diphosphatidylglycerol (DPG). The major respiratory quinone is ubiquinone 8 (Q-8). The DNA G + C content varies between 47.17 and 52.94 mol% (**Table 1**). The type species of the genus is *Pseudidiomarina taiwanensis*.

### Emended Description of *Pseudidiomarina taiwanensis* (Jean et al., 2006)

*Pseudidiomarina taiwanensis* (tai.wan.en’sis. N.L. fem. adj. *taiwanensis* pertaining to Taiwan, where the type strain was isolated).

The description is identical to that given for *Pseudidiomarina taiwanensis* by Jean et al. (2006). Additional characteristics are as follows: the polar lipids comprise PE, PG, DPG and seven uncharacterized phospholipids (PL1-PL6 and PL10); the respiratory quinone is Q-8 (Zhong et al., 2014). The type strain, PIT1^T^ ( = BCRC 17465^T^= JCM 13360^T^), was isolated from shallow coastal water of An-Ping Harbour, Taiwan. The DNA G + C content of the type strain is 47.35 mol%.

### Emended Description of *Pseudidiomarina homiensis* (Kwon et al., 2006; Jean et al., 2009)

*Pseudidiomarina homiensis* (ho.mi.en’sis. N.L. fem. adj. *homiensis* referring to the Homi Cape in Korea, where the type strain was isolated).

The description is identical to that given for *Idiomarina homiensis* by Kwon et al. (2006). Additional characteristics are as follows: the polar lipids comprise PE, PG, DPG, phosphatidylserine (PS), an uncharacterized aminolipid (AL1) and eight uncharacterized PLs (PL1-PL8) (Chen et al., 2012). The type strain is PO-M2^T^ ( = KACC 11514^T^= DSM 17923^T^), which was isolated from seashore sand in Pohang, Korea. The DNA G + C content of the type strain is 49.98 mol%.

### Emended Description of *Pseudidiomarina salinarum* (Yoon et al., 2007; Jean et al., 2009)

*Pseudidiomarina salinarum* (sa.li.na’rum. L. gen. pl. n. *salinarum* of salt-works).

The description is identical to that given for *Idiomarina salinarum* by Yoon et al. (2007). Additional characteristics are as follows: the polar lipids comprise PE, PG, DPG and six uncharacterized PLs (PL1-PL5 and PL11); the enzyme activities of *α*-mannosidase in API ZYM tests and D-glucose fermentation in API 20NE are variable (Zhong et al., 2014). The type strain, ISL-52^T^ ( = KCTC 12971^T^= CCUG 54359^T^), was isolated from a marine solar saltern of the Yellow Sea, Korea. The DNA G + C content of the type strain is 52.94 mol%.

### Emended Description of *Pseudidiomarina sediminum* (Hu and Li, 2007)

*Pseudidiomarina sediminum* (se.di’mi.num. L. gen. pl. n. *sediminum* of sediments, pertaining to source of isolation of the type strain).

The description is identical to that given for *Pseudidiomarina sediminum* by Hu and Li (2007). Additional characteristics are as follows: the polar lipids comprise PE, PG, DPG, PS, three uncharacterized ALs (AL1-AL3) and three uncharacterized PLs (PL4-PL6) (Chen et al., 2012). The type strain is c121^T^ ( = CICC 10319^T^= LMG 24046^T^), isolated from coastal sediment. The DNA G + C content of the type strain is 50.35 mol%.

### Emended Description of *Pseudidiomarina marina* (Jean et al., 2009)

*Pseudidiomarina marina* (ma.ri’na. L. fem. adj. *marina* of the sea, marine).

The description is identical to that given for *Pseudidiomarina marina* by Jean et al. (2009). Additional characteristics are as follows: the polar lipids comprise PE, PG, DPG, two uncharacterized ALs (AL1–AL2), an uncharacterized PL, and three lipids (L1–L3) (Poddar et al., 2014). The type strain, PIM1^T^ ( = BCRC 17749^T^= JCM 15083^T^), was isolated from shallow coastal seawater of An-Ping Harbour, Tainan, Taiwan. The DNA G + C content of the type strain is 47.17 mol%.

### Emended Description of *Pseudidiomarina tainanensis* (Jean et al., 2009)

*Pseudidiomarina tainanensis* (tai.nan.en’sis. N.L. fem. adj. *tainanensis* pertaining to Tainan, Taiwan, where the type strain was isolated).

The description is identical to that given for *Pseudidiomarina tainanensis* by Jean et al. (2009). Additional characteristics are as follows: the polar lipids comprise PE, PG, DPG, two uncharacterized ALs (AL1–AL2), an uncharacterized PL, and L1 (Poddar et al., 2014). The type strain, PIN1^T^ ( = BCRC 17750^T^= JCM 15084^T^), was isolated from shallow coastal seawater of An-Ping Harbour, Tainan, Taiwan. The DNA G + C content of the type strain is 47.35 mol%.

### Emended Description of *Pseudidiomarina donghaiensis* (Wu et al., 2009)

*Pseudidiomarina donghaiensis* (dong.hai.en’sis. N.L. fem. adj. *donghaiensis* pertaining to Donghai, the Chinese name for the East China Sea).

The description is identical to that given for *Pseudidiomarina donghaiensis* by Wu et al. (2009). Additional characteristics are as follows: the polar lipids comprise PE, PG, DPG, an uncharacterized AL1, an uncharacterized PL, and L1 (Poddar et al., 2014). The type strain, 908033^T^ ( = CGMCC 1.7284^T^= JCM 15533^T^), was isolated from a coastal seawater sample collected from the East China Sea, China. The DNA G + C content of the type strain is 48.07 mol%.

### Emended Description of *Pseudidiomarina maritima* (Wu et al., 2009)

*Pseudidiomarina maritima* (ma.ri’ti.ma. L. fem. adj. *maritima* inhabiting marine environments).

The description is identical to that given for *Pseudidiomarina maritima* by Wu et al. (2009). Additional characteristics are as follows: the polar lipids comprise PE, PG, DPG, an uncharacterized AL, an uncharacterized PL and two Ls (L1-L2) (Poddar et al., 2014). The type strain, 908087^T^ ( = CGMCC 1.7285^T^= JCM 15534^T^), was isolated from a coastal seawater sample collected from the East China Sea, China. The DNA G + C content of the type strain is 47.38 mol%.

### Description of *Pseudidiomarina insulisalsae* comb. nov.

*Pseudidiomarina insulisalsae* (in.su.li.sal’sae. L. fem. n. *insula*, island; L. adj. *salsus* -*a* -*um*, salted; N.L. gen. n. *insulisalsae*, of a salted island, isolated from the Island of Sal [the salt island] in the Cape Verde Archipelago).

Basonym: *Idiomarina insulisalsae* (Taborda et al., 2009)

The description is identical to that given for *Idiomarina insulisalsae* by Taborda et al. (2009). The type strain, CVS-6^T^ ( = CIP108836^T^= LMG 23123^T^), was isolated from the soil of a solar saltern. The DNA G + C content of the type strain is 52.33 mol%.

### Emended Description of *Pseudidiomarina aestuarii* (Park et al., 2010)

*Pseudidiomarina aestuarii* (aes.tu.a’ri.i. L. gen. n. *aestuarii* of a tract overflowed at high tide, a salt marsh, a bay, referring to the isolation of the type strain from shallow coastal seawater).

The description is identical to that given for *Pseudidiomarina aestuarii* by Park et al. (2010). Additional characteristics are as follows: the polar lipids comprise PE, PG, DPG, and six uncharacterized PLs (PL1-PL5, PL12); hydrolysis of urea, gelatin, L-arginine, and the enzyme activities of leucine arylamidase and trypsin in API ZYM tests are variable (Zhong et al., 2014). The type strain is KYW314^T^ ( = KCTC 22740^T^= JCM 16344^T^), isolated from seawater collected from the South Sea, South Korea. The DNA G + C content of the type strain is 49.10 mol%.

### Description of *Pseudidiomarina aquimaris* comb. nov.

*Pseudidiomarina aquimaris* (a.qui.ma’ris. L. fem. n. *aqua* water; L. gen. n. *maris* of the sea; N.L. gen. n. *aquimaris* of the water of the sea).

Basonym: *Idiomarina aquimaris* (Chen et al., 2012).

The description is identical to that given for *Idiomarina aquimaris* by Chen et al. (2012). The type strain, SW15^T^ ( = LMG 25374^T^ = BCRC 80083^T^), was isolated from a sample of a reef-building coral (*Isopora palifera*) collected off the coast of southern Taiwan. The DNA G + C content of the type strain is 50.24 mol%.

Description of *Pseudidiomarina indica* comb. nov.

*Pseudidiomarina indica* (in’di.ca. L. fem. adj. *indica* Indian, referring to the Indian Ocean, where the strain was isolated).

Basonym: *Idiomarina indica* (Song et al., 2013).

The description is identical to that given for *Idiomarina indica* by Song et al. (2013). The type strain, SW104^T^ ( = CGMCC 1.10824^T^ = JCM 18138^T^), was isolated from a seawater sample collected from the India Ocean. The DNA G + C content of the type strain is 49.30 mol%.

### Description of *Pseudidiomarina planktonica* comb. nov.

*Pseudidiomarina planktonica* (plank.to’ni.ca. N.L. fem. adj. *planktonica* (from Gr. adj. *planktos* wandering) living in the plankton, planktonic.)

Basonym: *Idiomarina planktonica* (Zhong et al., 2014).

The description is identical to that given for *Idiomarina planktonica* by Zhong et al. (2014). The type strain, TS-T11^T^ ( = CGMCC 1.12458^T^ = JCM 19263^T^), was isolated from a water sample of Tuosu lake in the Qaidam basin, Qinghai province, China. The DNA G + C content of the type strain is 48.97 mol%.

### Description of *Pseudidiomarina woesei* comb. nov.

*Pseudidiomarina woesei* (woe’se.i. N.L. gen. n. *woesei*, of Woese, named after Professor Carl R. Woese, a pioneer in evolutionary microbiology).

Basonym: *Idiomarina woesei* (Poddar et al., 2014)

The description is identical to that given for *Idiomarina woesei* by Poddar et al. (2014). The type strain, W11^T^ ( = DSM 27808^T^= JCM 19499^T^= LMG 27903^T^), was isolated from the Andaman Sea. The DNA G + C content of the type strain is 47.80 mol%.

### Description of *Pseudidiomarina halophila* comb. nov.

*Pseudidiomarina halophila* (ha.lo’phi.la Gr. n. *hals, halos* salt; N.L. fem. adj. *phila* (from Gr. fem. adj. *philê*), friend, loving; N.L. fem. adj. *halophila* salt-loving.)

Basonym: *Idiomarina halophila* (Lee et al., 2015).

The description is identical to that given for *Idiomarina halophila* by Lee et al. (2015). The type strain, BH195^T^ ( = KACC 17610^T^ = NCAIM B 02544^T^), was isolated from the sediment of a solar saltern in Gomso, Buan-gun, Chunbuk, South Korea. The DNA G + C content of the type strain is 50.58 mol%.

### Description of *Pseudidiomarina atlantica* comb. nov.

*Pseudidiomarina atlantica* (at.lan’ti.ca. L. fem. adj. *atlantica*, referring to the Atlantic Ocean, where the strain was isolated).

Basonym: *Idiomarina atlantica* (Du et al., 2015).

The description is identical to that given for *Idiomarina atlantica* by Du et al. (2015). The type strain, G5_TVMV8_7^T^ ( = MCCC 1A10513^T^= KCTC 42141^T^), was isolated from the deep sea sediment of the North Atlantic Ocean.

## Author Contributions

YL and ZS conceived and designed the experiments. YL performed the experiments, analyzed the data, and wrote the paper. YL, QL, and ZS revised the paper.

## Conflict of Interest Statement

The authors declare that the research was conducted in the absence of any commercial or financial relationships that could be construed as a potential conflict of interest.

## Footnotes

1 http://www.bacterio.net/

2 http://www.patricbrc.org

3 http://itol.embl.de/

4 http://ggdc.dsmz.de/distcalc2.php

5 https://www.ezbiocloud.net/tools/orthoaniu

## Supplementary Material

FIGURE S1 Boxplot of genomic G + C contents (left panel) and genomic sizes (right panel) of bacteria from the eight families within the order *Alteromonadales*. In the boxplot, the central rectangle spans the first quartile to the third quartile, a segment inside the rectangle shows the median, “whiskers” above and below the box shows the locations of the minimum and maximum, and the unfilled circles show suspected outliers. Means with different letters at the top of figure denote significant differences (Tukey’s hsd *post hoc* test, *α* = 0.05; a < b < c < d).

FIGURE S2 The supertree based on 16S rRNA gene sequences of 717 bacteria within the family *Idiomarinaceae*. The phylogeny was inferred by using FastTree 2.1.10 with JTT + CAT parameters and 1000 bootstrap replicates and rooted by using *E. coli* str. K-12 substr. MG1655. Bootstrap values are indicated on the nodes with different sizes of solid circle filled by light blue. Each of type strains were marked by a superscripted capital T.

TABLE S1 The detailed information of 92 *Idiomarinaceae* genomes used in this study.

TABLE S2 The 78 single-copy orthologous proteins used for tree inference.

TABLE S3 The pairwise similarites (%) of 16S rRNA gene sequences of 43 strains within the family *Idiomarinaceae*.

TABLE S4 The signature sequences of 16S rRNA gene for 48 strains within the family *Idiomarinaceae*.

TABLE S5 The pairwise dDDH values (%) between the 79 strains used in this study.

TABLE S6 The pairwise ANI values (%) between the 79 strains used in this study.

TABLE S7 The pairwise dDDH values (%) between the representative strains and the other thirteen strains marked by asterisks.

TABLE S8 The accession numbers of 16S rRNA gene sequences for 674 non-type strains within the family *Idiomarinaceae* from the GenBank database.
