# Supplementary figures and images for: Genome-Based Analysis Reveals the Taxonomy and Diversity of the Family Idiomarinaceae
Source: Front Microbiol. 2018 Oct 11;9:2453. doi: 10.3389/fmicb.2018.02453 (PMC6193092; doi:10.3389/fmicb.2018.02453)

Tree scale: 0.01

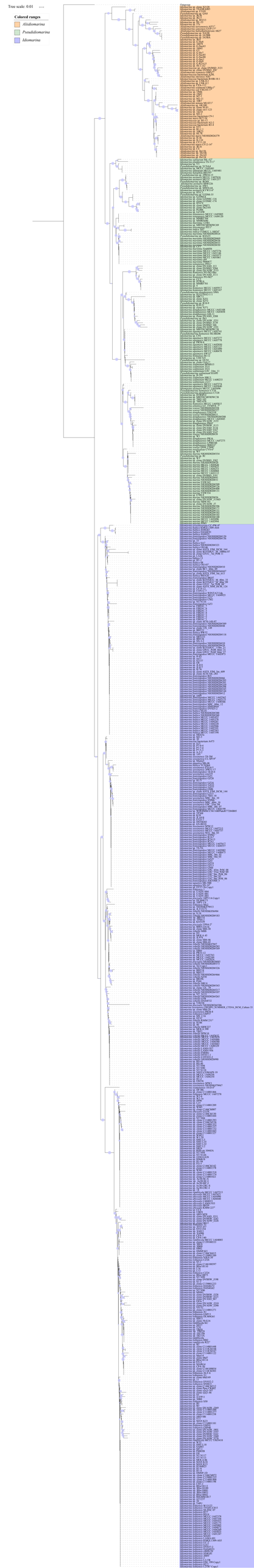

Supplement: FIGURE S1 — Boxplot of genomic G + C contents (left panel) and genomic sizes (right panel) of bacteria from the eight families within the order Alteromonadales. In the boxplot, the central rectangle spans the first quartile to the third quartile, a segment inside the rectangle shows the median, “whiskers” above and below the box shows the locations of the minimum and maximum, and the unfilled circles show suspected outliers. Means with different letters at the top of figure denote significant differences (Tukey’s hsd post hoc test, α = 0.05; a < b < c < d). [file Image_1.pdf]

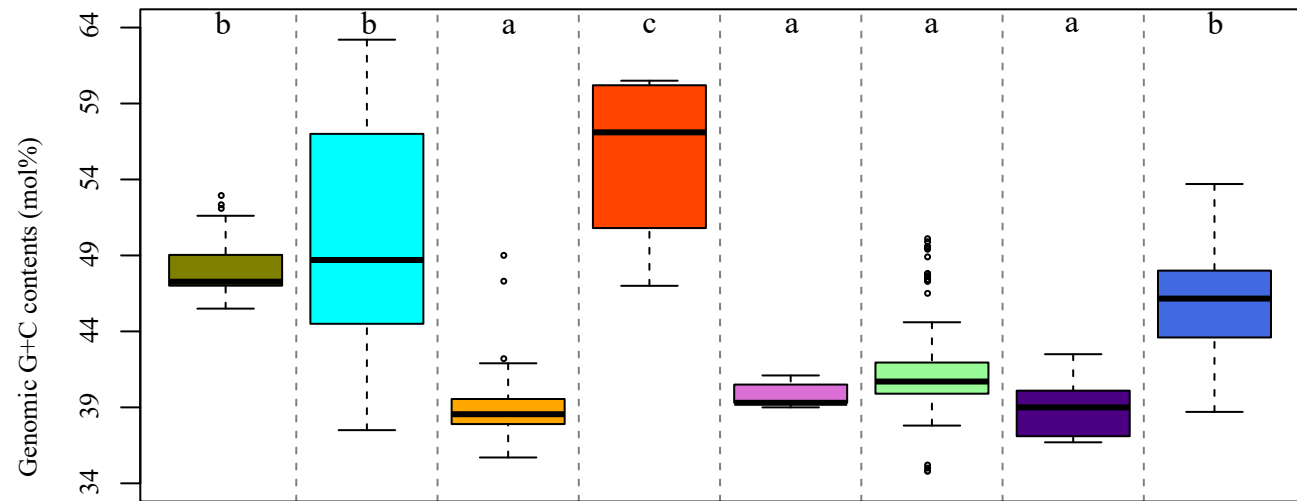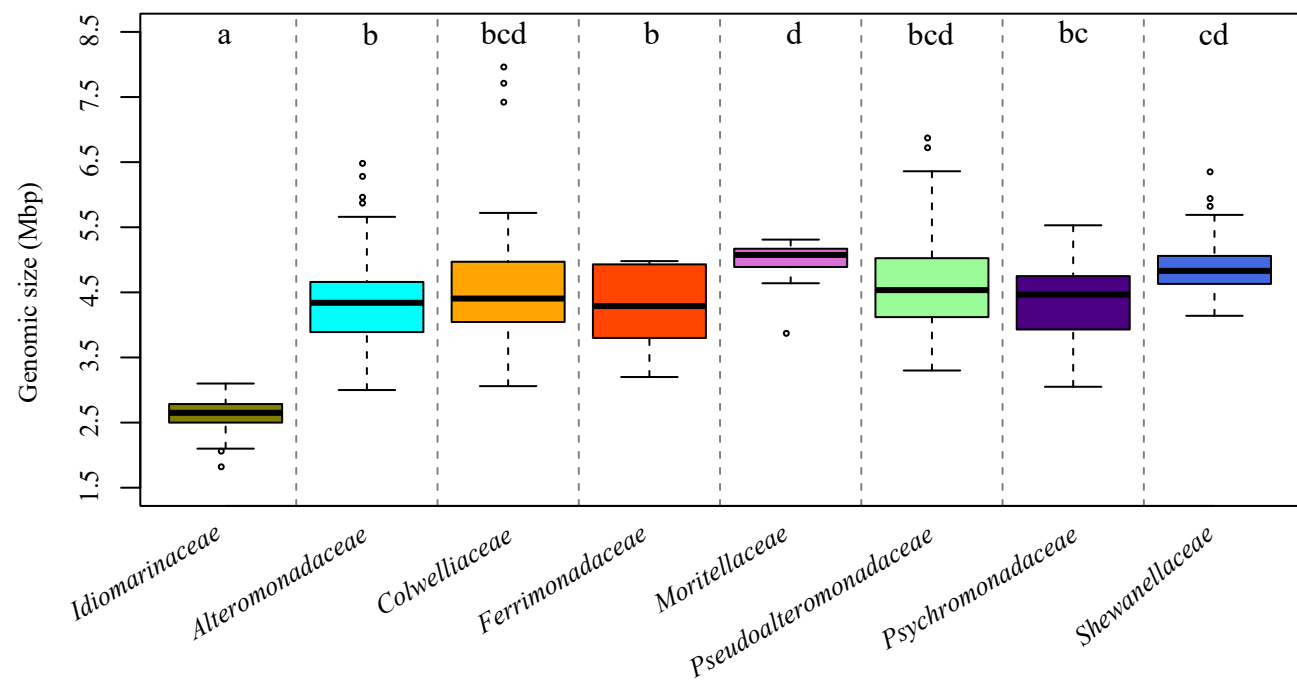

Supplement: FIGURE S2 — The supertree based on 16S rRNA gene sequences of 717 bacteria within the family Idiomarinaceae. The phylogeny was inferred by using FastTree 2.1.10 with JTT + CAT parameters and 1000 bootstrap replicates and rooted by using E. coli str. K-12 substr. MG1655. Bootstrap values are indicated on the nodes with different sizes of solid circle filled by light blue. Each of type strains were marked by a superscripted capital T. [file Image_2.pdf]
